# Flexural Strength of Internally Stiffened Tubular Steel Beam Filled with Recycled Concrete Materials

**DOI:** 10.3390/ma14216334

**Published:** 2021-10-23

**Authors:** Ahmed W. Al Zand, Mustafa M. Ali, Riyadh Al-Ameri, Wan Hamidon Wan Badaruzzaman, Wadhah M. Tawfeeq, Emad Hosseinpour, Zaher Mundher Yaseen

**Affiliations:** 1 Department of Civil Engineering, Universiti Kebangsaan Malaysia (UKM), Bangi 43600, Malaysia; p86577@siswa.ukm.edu.my (M.M.A.); wanhamidon@ukm.edu.my (W.H.W.B.); emadhoseinpour@gmail.com (E.H.); 2 Department of Civil Engineering, University of Technology, Baghdad 10066, Iraq; 3 School of Engineering, Faculty of Science Engineering and Built Environment, Deakin University, Geelong, VIC 3216, Australia; r.alameri@deakin.edu.au; 4 Faculty of Engineering, Sohar University, Sohar 311, Oman; WTawfeeq@su.edu.om; 5 New Era and Development in Civil Engineering Research Group, Scientific Research Center, Al-Ayen University, Thi-Qar 64001, Iraq; yaseen@alayen.edu.iq

**Keywords:** stiffened CFST beam, recycled concrete, finite element, flexural strength, cold-formed tube

## Abstract

The flexural strength of Slender steel tube sections is known to achieve significant improvements upon being filled with concrete material; however, this section is more likely to fail due to buckling under compression stresses. This study investigates the flexural behavior of a Slender steel tube beam that was produced by connecting two pieces of C-sections and was filled with recycled-aggregate concrete materials (CFST beam). The C-section’s lips behaved as internal stiffeners for the CFST beam’s cross-section. A static flexural test was conducted on five large scale specimens, including one specimen that was tested without concrete material (hollow specimen). The ABAQUS software was also employed for the simulation and non-linear analysis of an additional 20 CFST models in order to further investigate the effects of varied parameters that were not tested experimentally. The numerical model was able to adequately verify the flexural behavior and failure mode of the corresponding tested specimen, with an overestimation of the flexural strength capacity of about 3.1%. Generally, the study confirmed the validity of using the tubular C-sections in the CFST beam concept, and their lips (internal stiffeners) led to significant improvements in the flexural strength, stiffness, and energy absorption index. Moreover, a new analytical method was developed to specifically predict the bending (flexural) strength capacity of the internally stiffened CFST beams with steel stiffeners, which was well-aligned with the results derived from the current investigation and with those obtained by others.

## 1. Introduction

The usefulness of various types of concrete-filled steel tube (CFST) has been proven in modern composite structural projects since they have achieved better performances (in terms of strength, ductility and stiffness) than the corresponding conventional concrete/steel members under axial and flexural loading [1,2,3,4]. In general, the structural performance of concrete-filled steel composite members, under different loading scenarios, has been experimentally and numerically examined in several studies; for example, those presented in [5,6,7,8,9].

Particularly, the adequacy of using the Slender steel tube section (Class 4; as per Eurocode classification) in the concept of CFST beams has been previously investigated [10,11,12,13,14]. This section (Slender) has achieved more strength improvement percentages than those of the Noncompact and Compact sections, after being filled with concrete materials, as compared to their corresponding hollow tube sections [10,15]. In addition, the steel tubes of CFST members with Slender cross-sections usually have lighter self-weight than the Noncompact and Compact sections; thus, they are more favourable in terms of reducing the impact of cost in construction projects. However, the Slender tube section is more likely to buckle outward at the compression zone stress because of Slender structural sections [16,17]. Therefore, internal steel stiffeners were provided to delay and restrict the outward tube’s buckling of the Slender CFST beams’ cross-section [18,19,20,21]. For example, Al Zand et al. [19] experimentally and numerically examined the effect of using varied shapes of internal steel stiffeners that were welded along all sides of high-slenderness cold-formed square CFST beams. Their investigation showed that these CFST beams’ flexural stiffnesses and strength capacities were improved by about 22–29% when a single stiffener was welded along each tube’s internal sides, and these values were increased by a further 48–59% when double steel stiffeners were provided. Furthermore, in some cases, the CFST beam’s cross-sections were externally stiffened, either by using additional steel plates that were fixed mechanically along the beam’s flanges [5], or by preparing a cold-formed tube’s cross-section with V-grooves that were provided along the sides of the CFST beam [22]. However, the welding of additional steel stiffeners for the stiffening of the CFST beam’s cross-section could require an additional fabrication process and extra labour costs. Thus, using pre-fabricated cold-formed tubular steel sections (C-sections/C-Purlins) without extra welding and/or mechanical fixation could be considered as a new concept for use in the stiffened Slender CFST beam system, particularly for lightweight flooring structures. For example, in Malaysia, the cost of 1 ton of fabricated steel sections is about 1000 USD, and the equivalent cost of hot-rolled sections is about 1200 USD, while the cost of 1 ton of concrete is about 40–50 USD. This material’s cost comparison shows that it is very useful if the engineers can utilise CFST beams that have been prepared from cold-formed sections filled with concrete in the lightweight composite structures, even though the self-weight of these composite beams can increase by 5–8 times as compared to the equivalent steel beams due to the effect of concrete infill material.

The research topic of CFST has received considerable attention over the past few years from a wide range of structural engineering scholars. A dependent stress–strain model was developed for the CFST column [23]. The flexural behaviour of steel-fibre-reinforced self-stressing recycled-aggregate CFST was studied by [24]. The behaviour of heated CFST stub columns, containing steel fibre and tire rubber, was tested by [25]. Reinforced CFST with steel bars was studied experimentally in terms of its seismic behaviour [26]. Several other related research studies on the advanced application of CFST can be reviewed through [27,28]. Nevertheless, this review of the literature evidenced the importance of the enhancement of concrete properties for the achievement of better reliability and robustness in construction projects [29,30,31].

In recent years, engineers have been increasingly utilizing the recycled aggregate generated from the demolition of existing materials and waste materials in the field of construction [24,32,33,34]. There have been several studies on the behaviour of Recycled-Aggregate Concrete (RAC) material, which is prepared by crushing the old structural elements of concrete. For example, variations in the RAC replacement percentages (0%, 25% and 50% of the raw aggregate) were adopted in the concrete infill material for CFST members under flexural and compression loads [11,24,35,36,37,38,39,40]. The results achieved were quite similar to those obtained for CFST members filled with normal concrete, albeit with slightly lower strength capacities [38]. Recently, Liu et al. [24] used RAC and sulphoaluminate cement in varied concrete mixtures, which were reinforced with steel fibre and used as infilling material for 54 CFST beams. Their experimental study reveals that the reinforcement of the recycled-aggregate concrete mixture with 1.2% steel fibre can achieve similar flexural behaviour to that of the CFST beams that are filled with normal concrete. Furthermore, using the lightweight concrete infill material can significantly reduce the overall self-weight of the CFST members [17,41,42,43], which is significant given that the self-weight represents one of the most important challenges in terms of adopting composite members of this type in modern structures. Generally, using expanded polystyrene (EPS) beads as a replacement for the raw aggregate is the most effective method that is usually used to achieve lightweight concrete mixtures [19,44,45,46]. For instance, EPS can also be sourced from waste polystyrene materials (recycled materials). However, the performance of cold-formed steel tubes (the Slender tube section) filled with lightweight and recycled concrete material under pure flexural loading has not yet been extensively investigated. In such cases, as explained earlier, the overall self-weight and the cost impact constitute important factors in the Engineer’s decision to adopt this system in modern composite structures. Therefore, at present, finding an alternative CFST composite structural design concept is a matter of urgency.

The main objectives of this research can be highlighted as follows: The first aim was to examine the suitability of using the new concept of cold-formed tubular cross-sections fabricated from two pieces of galvanized C-sections (face-to-face connection) in the Slender CFST beam’s system, in which the lips of these C-sections are expected to behave as internal steel stiffeners along the top and bottom flanges. Second, this research project aimed to explore the behaviour of the proposed Slender CFST beams, specifically when recycled-aggregate concrete mixture is used instead of the normal concrete mixture, in order to reduce the self-weight and cost of the beam’s section. Lastly, to date, all of the theoretical methods for predicting the flexural strength of CFST beams were developed according to different standards, and they were mostly intended to be applied for beams with conventional tube sections (unstiffened sections). As such, this study aimed to develop a new analytical method that can theoretically predict the flexural strength capacity (ultimate bending moment; *M_u_*) of the internally stiffened CFST beam’s cross-section with varied numbers/sizes of steel stiffeners; this constitutes one of the main novelties of the current research work. Therefore, five CFST specimens were prepared for this purpose and were experimentally tested under pure flexural loading, including one specimen without concrete filling material (hollow tube section). The finite element (FE) ABAQUS software was used to develop and analyse an additional 20 models that were designed to examine the effect of further parameters that were not explored experimentally.

## 2. Experimental Approach

### 2.1. Preparation of Samples

**Figure 1:**
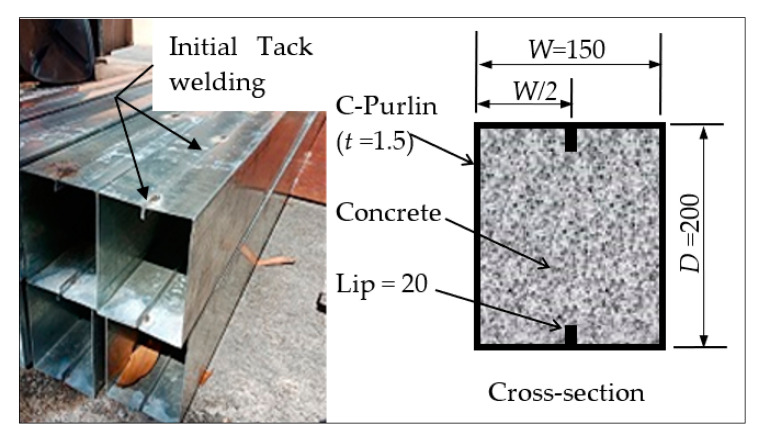
Cold-formed steel C-sections (all dimensions in mm).

In order to suggest a new concept for the design of Slender steel tube cross-sections that are internally stiffened, in this research, five specimens were produced from two steel C-sections connected by means of tack welding to obtain the suggested steel tubular section, as shown in Figure 1. These prefabricated tubular sections had 20 mm lips that functioned as internal stiffeners along the top and bottom flanges of the tubular beams. A single specimen was tested as a hollow tube beam (HB), while the other four tubes’ specimens were filled with different concrete mixtures (filled beam; FB). The raw coarse aggregate of the concrete mixtures was replaced with different recycled-aggregate materials (EPS and RAC), since one of the main objectives of this research was to reduce the self-weight and cost of the mixture. The replacement percentages of raw coarse aggregate (by volume) were equal to 0% (filled beam designation; FB-RC0), 30% (FB-RC30), 50% (FB-RC50), and 70% (FB-RC70), as presented in Table 1. All steel tubes specimens were placed vertically, after which the concrete material was poured from the top ends in multiple stages, while the bottom ends were temporary sealed to prevent water leakage.

### 2.2. Material Properties

Steel tube: Three coupons were cut from the cold-formed C-sections and prepared in accordance with the ASTM-E8/E8M-2009 standard. The average results of the yield tensile strength (*f_y_*), maximum elongation (%), ultimate tensile strength (*f_u_*), and elastic modulus (*E_s_*) were 489 N/mm^2^, 27.4%, 558 N/mm^2^, and 201 × 10^3^ N/mm^2^, respectively, and these results were obtained from the direct tensile of coupons test.

Concrete mixtures: the recycled aggregates used in the suggested concrete mixtures were EPS beads (4–6.3 mm) with a density of 9.5 (kg/m^3^), and RAC (4.75–16 mm) with a density of 1278 kg/m^3^; these densities were much lower than that of the raw coarse aggregate (1490 kg/m^3^). As explained earlier, the first concrete mixture was prepared without the use of any recycled aggregate (normal concrete; 0% replacement aggregate: RC0). The second concrete mixture (RC30) was prepared by replacing 30% (by volume) of the raw coarse aggregate by EPS beads only. Meanwhile, the concrete mixtures RC50 and RC70 were also made by replacing 30% of the raw aggregate with EPS beads and adding another 20 and 40% (in terms of total volume) of RAC, respectively, bringing the total replacement to 50 and 70%, respectively. In addition, silica fume (SF) material was used to enhance the bonding performance between the cement and EPS beads, as previously advised in [45]. Lastly, for all concrete mixtures, a water/cement (w/c) ratio of 0.46 was used together with the superplasticizer liquid (Real Flow 611). The proportions of the concrete mixtures are presented in Table 2. For each concrete mixture, three samples of concrete cubes (150 mm) were prepared, cured for up to 28 days, and tested in accordance with BS 1881: 1983.

### 2.3. Test Setup

**Figure 2:**
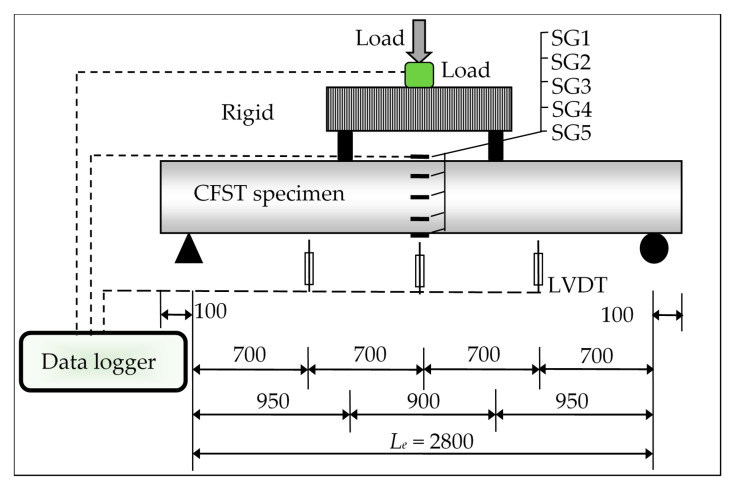
Schematic of specimens’ test setup (all dimensions in mm).

All specimens were tested under four-point static loads, as shown in the schematic of the test setup in Figure 2. A hydraulic jack with a maximum capacity of 500 kN was used to apply the static load on the suggested CFST specimens. Utilizing linear variable differential transducers (LVDTs) type KYOWA, Osaka, Japan, the vertical deflections of the specimens were recorded at different locations. The strain gauges SG1 to SG5 were fixed vertically at the mid-span of each CFST specimen. A data logger was used for collection of the data from the load cell, strain gauges, and LVDTs during the tests, and these data were recorded in a computerized system.

## 3. Discussion of Experimental Results

### 3.1. Failure Modes

**Figure 3:**
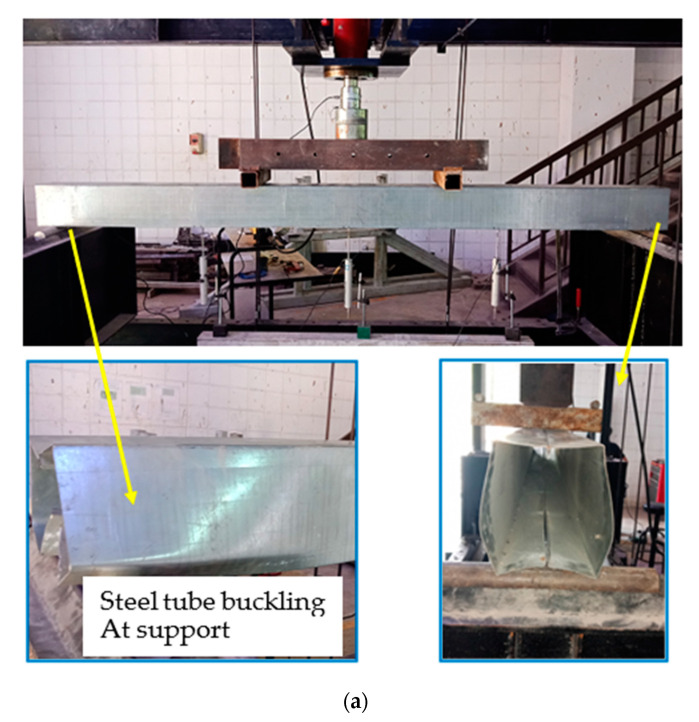

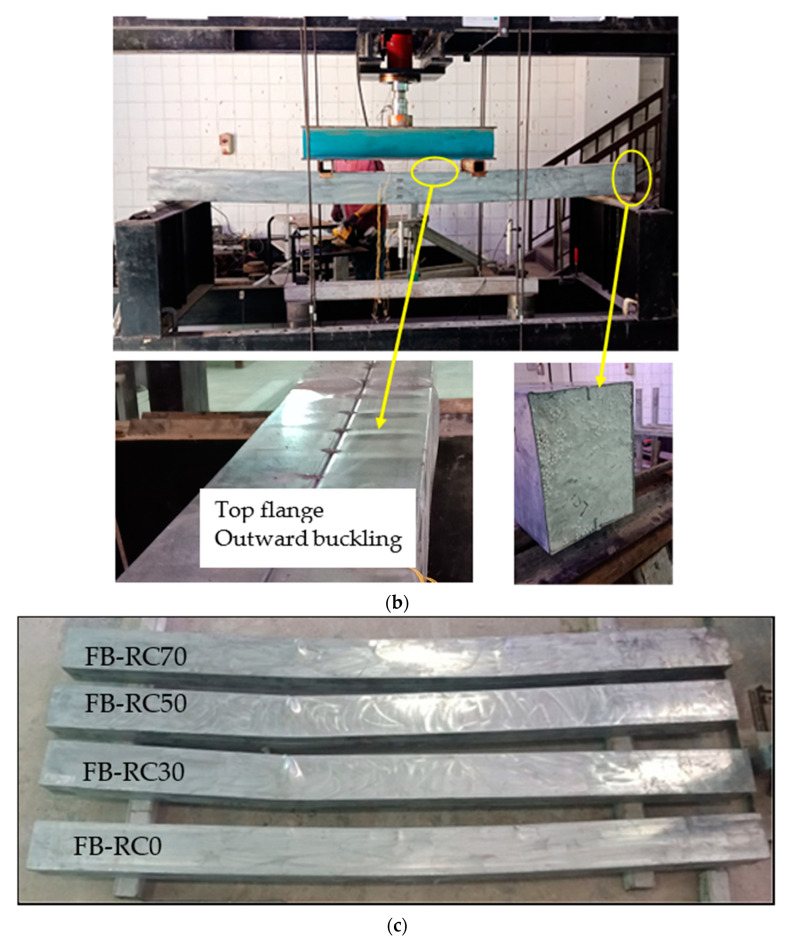
Typical failure modes: (**a**) unfilled specimen (HB); (**b**) filled specimen (FB-RC30); (**c**) all filled specimens after testing.

All specimens were tested beyond their strength capacities in order to study their extreme failure performance. The hollow steel tube specimen (HB) showed the inward tube’s buckling failure at the support points only, wherein the failure of tube’s webs was increased gradually by increasing the applied load, as shown in Figure 3a. Meanwhile, all filled specimens (FB-RC0, FB-RC30, FB-RC50, and FB-RC70) showed almost typical failure modes regardless of the types of their concrete mixtures. It was found that, for all filled specimens, outward buckling failure started occurring at the top tube’s flange, between the two-point loads of these filed specimens, particularly when the applied load values reached about 70–80% of their ultimate loading capacity, as shown in Figure 3b. This performance was due to the effects of the concrete infill materials, which mainly prevented the occurrence of inward buckling failure of the steel tube. Figure 3c presents all filled specimens after testing at the extreme failure limits.

**Figure 4:**
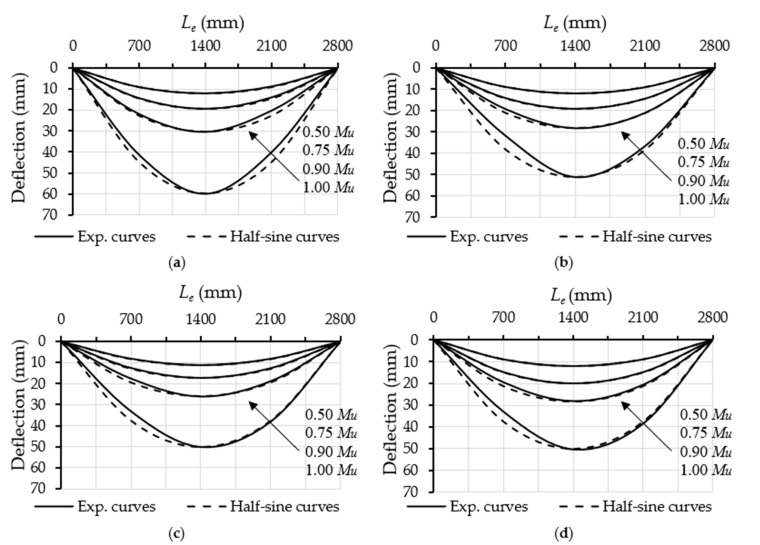
Deflection shapes of the filled specimens: (**a**) FB-RC0; (**b**) FB-RC30; (**c**) FB-RC50; (**d**) FB-RC70.

Furthermore, the 20 mm lips located at the top and bottom edges of the prefabricated cold-formed C-sections were well bonded with the concrete core, thus implying that these lips functioned sufficiently as internal stiffeners for the top and bottom flanges of the steel tubes of these filled specimens. Therefore, the outward buckling failure occurred only at the half-width of their top flanges (see Figure 3b). These lips achieved similar contributions to those of the additionally welded steel stiffeners that were included in previous research to stiffen the Slender CFST members [19,47]. Moreover, there was no slippage failure between the steel tube and the concrete core observed at both opened ends of the tested filled specimens. Unlike the unfilled specimen (HB), the filled specimens (FB-RC0, FB-RC30, FB-RC50 and FB-RC70) displayed a smooth deflection behaviour during the loading stages, which was quite similar to the half-sine curve, as illustrated in Figure 4. This performance allowed the CFST beams to distribute the point loads with almost uniform stress along the beam’s span, which was very similar to the beams that were loaded in a uniform loading scenario during the practical application of the members. Based on this finding, the above hypothesis was confirmed insofar as the currently suggested prefabricated cold-formed tubular steel beam filled with recycled concrete materials performed very similarly to the conversional CFST beams, which were tested earlier, for example, in [11,17,19,48].

### 3.2. Flexural Behaviour and Strength Capacity

**Figure 5:**
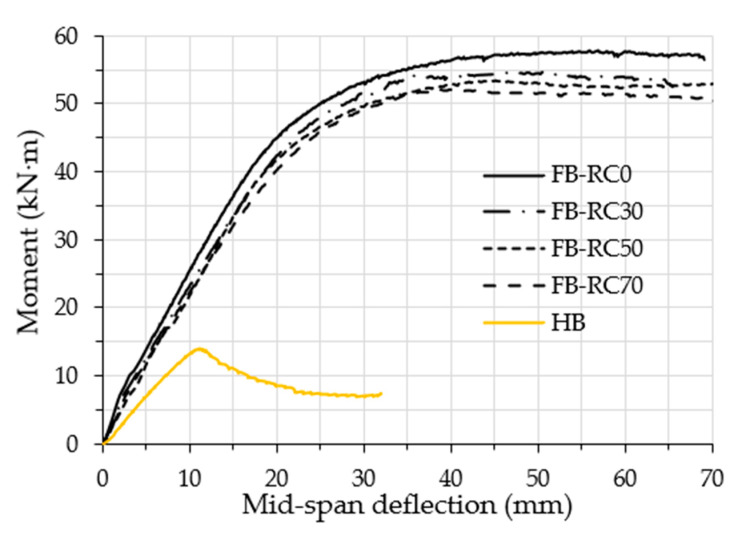
Moment vs. mid-span deflection relationships.

This section discusses the flexural behaviour, ultimate moment capacity (*M_u_*), and the moment vs. strain relationships of the tested specimens. The relationships between the bending moment and the deflection at mid-span for the tested specimens are compared in Figure 5, including the hollow specimen (HB). In general, for the concrete filled specimens, the moment–deflection curves continued to show linear behaviour until an archive of about 50–60% of their *M_u_* values, after which these curves behaved as elasto-plastics up to the loading limits of about 70–80% of their *M_u_* values (at this point, buckling failure of the top flange started to occur). Thus, due to further outward buckling failure, the same moment–deflection curves showed fully plastic behaviour with continual slow decreases. The *M_u_* value of the filled specimens was recorded at the deflection limit of *L_e_*/50 *− L_e_*/60 [49], which was about 46 mm to 56 mm. However, the moment–deflection curve of the unfilled specimen (HB) showed an almost linear behaviour until the peak loading point (*M_u_*) was achieved, at which the bottom flanges started to buckle inwardly at the supporting points; furthermore, as a result of extreme tube buckling failure, the loading curve began to descend.

The *M_u_* values of the tested specimens are presented in Table 1. The HB specimen achieved the ultimate flexural capacity of 14.1 kN·m; this capacity increased to 57.7 kN·m when the same fabricated steel tubular specimen was filled with normal concrete (FB-RC0), achieving an improvement of 409%. The tubular specimen filled with varied recycled concrete achieved *M_u_* values that were slightly lower than those of the FB-RC0 specimen (57.7 kN·m); these values were 53.7 kN·m, 52.6 kN·m and 51.5 kN·m, for specimens FB-RC30, FB-RC50 and FB-RC70, respectively. In addition, it was noticed that, regardless of the aggregate replacement percentages, the concrete filled tubular specimens achieved much higher flexural strength capacity values as compared to the capacity of the hollow specimen (HB), with a strength improvement of about 363 to 381%. It is worth mentioning that the self-weight of the tube beam was substantially increased due to the addition of the filled concrete; however, the flexural strength was remarkably enhanced, as stated above (363–409%). From the structural engineering point of view, this represents a very important finding regardless of the increment of the weight, and, in this case, the role of the structural engineer, who can prospectively determine scenarios in which this would be reliable for the targeted purpose of construction projects. On the other hand, the cost was also found to be a vital element in the construction design when we compared the concrete cost with the steel cost; this was the case because, in global terms, the cost of 1 ton of steel is about 15–20 times the cost of 1 ton of concrete material.

**Figure 6:**
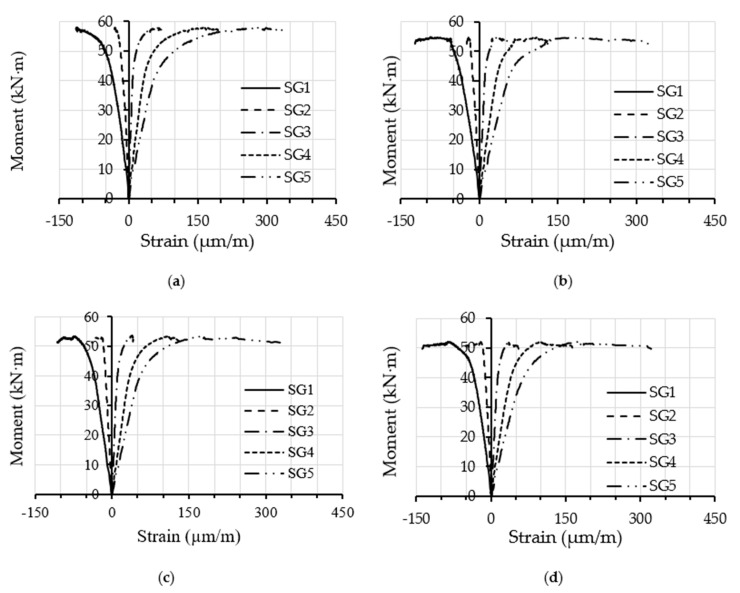
Moment vs. longitudinal strain relationships of the filled specimens: (**a**) FB-RC0; (**b**) FB-RC30; (**c**) FB-RC50; (**d**) FB-RC70.

**Figure 7:**
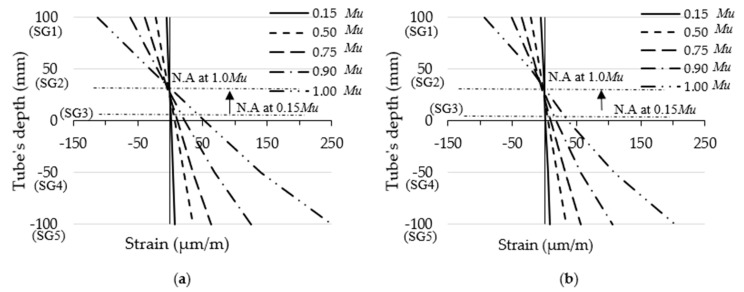
Depth of steel tube cross-section vs. longitudinal strain relationships: (**a**) FB-RC0; (**b**) FB-RC50.

Furthermore, the moment vs. longitudinal tensile strain relationships obtained at the mid-span of the steel tube of the filled specimens are presented in Figure 6. In this figure, it can be seen that the moment–strain relationships showed a fairly typical behaviour for all of the filled specimens regardless of the percentages of replacement aggregate in the concrete material [11,24]. The strain gauges SG1 and SG2 (see Figure 2) showed gradually increasing negative values (compression stress) with the increasing of the bending loads, while the strain gauges located at the bottom-half of beam’s cross-section (SG4 and SG5) showed gradual increases in their tension-related stress values. In contrast, the strain gauge SG3 (located in the middle of the specimen’s mid-span) showed a slight increase in tension-related stress, confirming that a positive correlation was discovered with the upward movement of the neutral axis in the tested filled specimens as the bending loads increased. Figure 7, as an example, shows the maximum strains distributed at the tube’s mid-span depth along its cross-section for specimens FB-RC0 and FB-RC30 during a variety of loading stages. In the current study, the aforementioned moment–strain relationships of the fabricated filled-steel tubular beams behaved similarly to those obtained for the conventional cold-formed CFST beams [11,48].

### 3.3. Flexural Stiffness

The initial stiffness (*K_i_*) and serviceability stiffness (*K_s_*) levels of tested specimens are usually measured from the moment vs. mid-span curvature relationships [50,51,52,53], which are based on moment values of 0.2*M_u_* and 0.6*M_u_*, respectively. The *K_i_* and *K_s_* values of the tested specimens are presented in Table 1. The HB specimen achieved the lowest flexural stiffness values as compared to those of the filled specimens (FB), which were 1207 kN·m^2^ and 1121 kN·m^2^ for *K_i_* and *K_s_*, respectively. These values increased to 2216 kN·m^2^ and 1924 kN·m^2^ for the FB-RC0 specimen due to the influence of the normal concrete infill material. In general, similar flexural stiffness improvement behaviours were recorded for all of the filled recycled concrete materials (FB-RC30, FB-RC50 and FB-RC7), but with slightly lower values than that of FB-RC0, since they had lower concrete modulus (*E_c_*) values. For example, compared to the FB-RC0 specimen (0% aggregate replacement), the FB-RC70 specimen, which was filled with recycled concrete (30% EPS plus 40% RAC) achieved lower *K_i_* and *K_s_* values (−6.7% and −12.1%, respectively). From the above discussion, it can be concluded that even when using the recycled aggregate in the concrete infill materials of the cold-formed CFST beams, the flexural stiffness can nevertheless be significantly improved, to a far greater degree than that of the corresponding hollow tube beam.

### 3.4. Energy Absorption Index

The ability of CFST beams to dissipate energy compared to that of the hollow steel tube beams was confirmed in several previous research studies [19,54]. Most commonly, the energy absorption index (EAI) of CFST beams was estimated from the cumulative area under the load vs. the deflection curves, up to the point at which the maximum beam strength limit had been reached [22,54,55]. The EAI values of the tested specimen in the current research are presented in Table 1; these values clearly confirm that the energy dissipation ability of the newly fabricated hollow specimen was extremely enhanced when filled with normal/recycled concrete materials. For example, the EAI value of the HB specimen increased from 201.8 kN·mm to 5996 kN·mm (29.7 times) when it was filled with normal concrete (FB-RC0). Moreover, compared to the tubular specimens that were filled with normal concrete (0% replacement aggregate), the specimens that were filled with recycled aggregate (EPS and RAC) had a slightly lower ability to dissipate energy, which can be considered a logical outcome since they achieved slightly lower loading capacities (see Figure 6). It is interesting to note that the EAI values of specimens FB-RC30, FB-RC50, and FB-RC70 were approximately 5539 kN·mm (−7.6%), 5493 kN·mm (−8.3%), and 439 kN·mm (−9.2%), respectively, as compared to the value of the FB-RC0 specimen (5996 kN·mm). These values represent significant achievements when compared to the value obtained for the corresponding unfilled HB specimen (201.8 kN·mm), being approximately 26.9 to 27.5 times higher

## 4. Numerical Approach

### 4.1. Development and Verification of the Numerical Model

**Figure 8:**
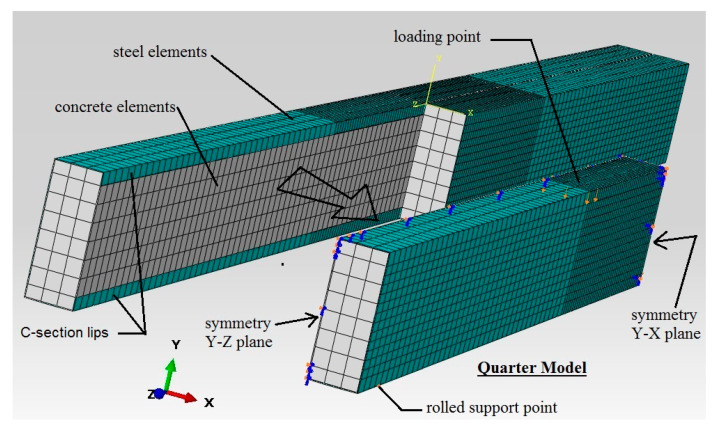
Typical finite element CFST model.

The suggested CFST beam was further investigated in terms of its flexural behaviour by using the ABAQUS 6.14 software to conduct non-linear finite element (FE) analysis. In order to reduce the time conception of the analyses of the models, a typical 3D quarter model was built to simulate the actual tested CFST specimen, as shown in Figure 8, which had the advantages of the symmetric cross-section and the beam’s loading scenario already having been prepared [15,19,56,57,58]. The actual test loading scenario was implemented in the FE modelling scenario by allowing the nodes that were positioned at the upper tube’s flange (loading point) to gradually move downwards during the FE analysis, using the incremental downward displacement option (displacement control approach), which is available in the software. The nodes placed at the support’s location were restricted from horizontal and vertical movement, but they were allowed to freely rotate around the X-axis to simulate the roller support. Then, the loading value of the FE model was obtained from the reaction forces that were observed at the support’s nodes [19,56].

The main component materials of the currently developed FE CFST models are the concrete infill and the steel tube (double C-sections). For the concrete component, the C3D8R element type was used, which has eight nodes integrated with six degrees of freedom, while, for the steel tube components, the type S4R shell element was used. The penalty friction coefficient of 0.75 was adopted in the current FE analysis to realise the mechanical interaction between the surfaces of the steel and concrete parts. In general, several parameters that mainly affected the proper friction coefficient values, such as the loading type, the size/shape of the beam’s cross-section, and the properties of the materials were selected [10,19,56,57,58]. Thus, in the current study, a convergence study was adopted in order to establish a suitable friction coefficient value (0.75), where several preliminary FE CFST models, which had varied friction coefficient values ranging from 0.4 to 0.9, were utilised. A finer mesh size was used for the distance between the two applied loads to sufficiently represent the failure modes of the analysed CFST models.

Consequently, the material properties of the steel tube and concrete components of the FE model were the same as those of the corresponding tested specimen. Generally, the concrete material is considered to be a brittle material since it cracks under tension-related stress and is crushed under compression stress [15]. Thus, the “Concrete Damage Plasticity” approach was adopted for the current FE models in order to ensure the compressive and tensile performance of the concrete infill component [15,57,59,60]. The elastic modulus and Poisson’s ratio of the steel component were identified in the elastic-isotropic section, while the plastic-isotropic option was used to identify the steel-yielding strength and the relevant strain values. For both the steel and concrete materials of the developed FE CFST models, the constitutive stress vs. strain relationships were estimated by adopting the same expressions that were used earlier in [15,56].

**Figure 9:**
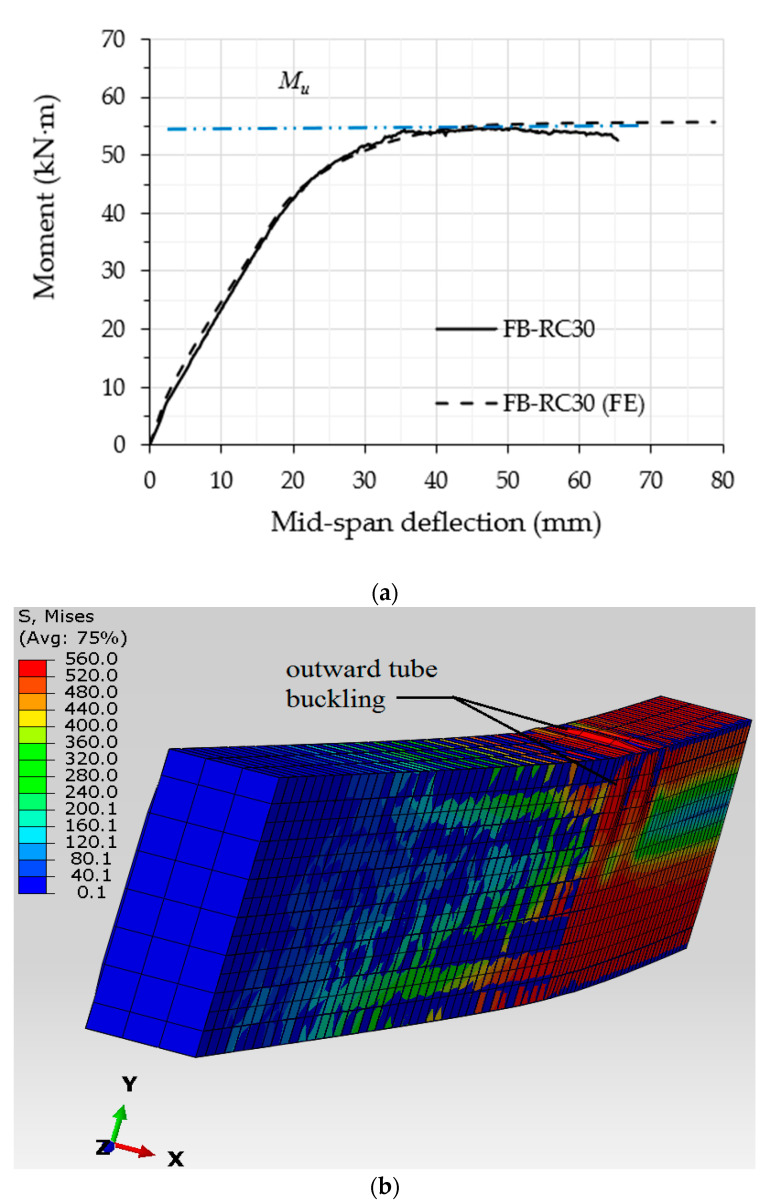
Verification of the FB-RC30 FE model with the corresponding tested specimen: (**a**) flexural behavior; (**b**) numerical failure mode.

The validity of the current FE models was confirmed via the corresponding experimental results; almost all of the filled specimens with varied concrete mixtures achieved similar behaviours and close flexural strength capacities. Thus, the FB-RC30 specimen (a steel tubular beam filled with a recycled concrete mixture of 30% EPS beads) was selected to verify the relevant FE model. The numerical analysis showed that the moment vs. mid-span deflection relationship of the FB-RC30 (FE) model sufficiently agreed with that of the corresponding tested specimen (FB-RC30), as shown in Figure 9a. The flexural strength capacity value of the numerical model of FB-RC30 was overestimated by about 3.1% (55.4 kN·m) as compared to that obtained from the corresponding tested specimen, which was an acceptable degree of deviation. In addition, the outward buckling failure occurring at the top-half cross-section of the tested FB-RC30 specimen, for the distance between the two-point loads, was numerically simulated by the relevant FE model, as shown in Figure 9b.

### 4.2. Parametric Studies

After confirming the validity of the developed CFST model, additional models were built and analysed to investigate the influence of further parameters. In particular, the effects of varied tube thickness (1.0 mm to 3.0 mm), concrete infill strength (14.6 MPa to 55.0 MPa), steel yield strength (275 MPa to 550 MPa), and steel tube depth (150 mm to 250 mm) were studied, and these were categorized into groups A, B, C and D, respectively. Table 3 presents the model’s designation along with the physical properties adopted for the 20 CFST models, including the *M_u_*, *K_i_* and *K_s_* values derived from their FE analyses.

#### 4.2.1. Performance of Bending Behaviour

**Figure 10:**
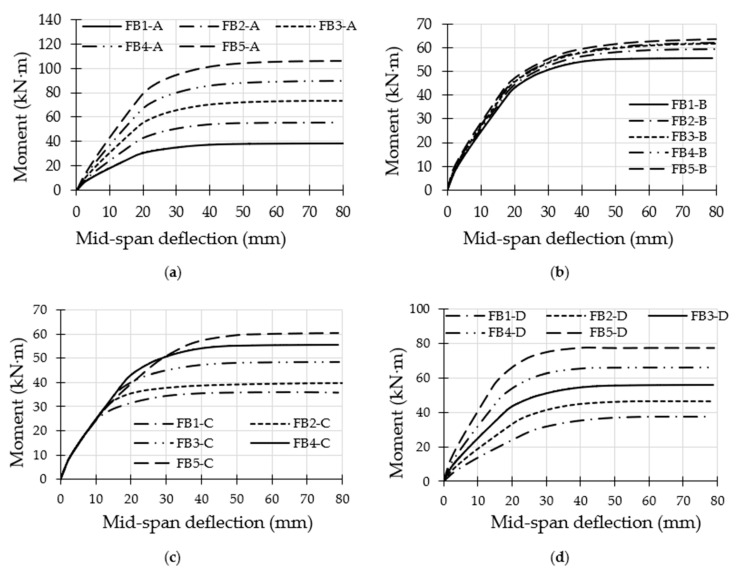
Moment vs. mid-span deflection of CFST models with varied parameters: (**a**) group A; (**b**) group B; (**c**) group C; (**d**) group D.

In Figure 10, the moment vs. mid-span deflection curves of the analysed FE models are presented independently for each group. These curves showed elastic behaviour at the initial loading stage up to a certain limit, followed by plastic behaviour until the ultimate strength capacity of the CFST model was achieved. The models with varied tube thicknesses and depths showed a major influence on their moment–deflection curves at both the elastic and plastic loading stages, as shown in Figure 10a,d for the FE models in groups A and D, respectively. This is a logical flexural behaviour since the cross-section parameters (the steel area and moment of inertia) of the suggested CFST models were increased as a result of enhancements in their tubes’ thicknesses and/or depths. However, the use of varied tube yield strengths did not bring about a major impact on the models’ moment–deflection behaviours at the elastic stage, but a major effect was found at their plastic stage only, as shown in Figure 10c for the models in group C. Moreover, very limited improvement was recorded for the moment–deflection curves’ behaviour when only the compressive strength of the infill material increased, as shown in Figure 10b.

#### 4.2.2. Performance of Stiffness

The stiffness values of the analysed FE models are given in Table 3. The *K_i_* and *K_s_* values improved significantly with the increases in the steel tube’s thickness and/or the depth of the studied models (group A and D), while very limited improvements were recorded when only the concrete compressive strength was increased (models in Group C). For example, the FB2-A model achieved *K_i_* and *K_s_* values of 2375 kN·m^2^ and 2125 kN·m^2^, respectively. These values were improved by about 50–55% (3575 kN·m^2^ and 3285 kN·m^2^) when only the tube’s thickness increased from 1.5 mm to 3.0 mm (FB5-A). Moreover, the stiffness *K_i_* and *K_s_* values of the FB1-B model were improved by about 18–19% (2819 kN·m^2^ and 2529 kN·m^2^) when only the ***f_cu_*** value increased by about three times (from 14.6 MPa to 45 MPa).

#### 4.2.3. Performance of Bending Strength

**Figure 11:**
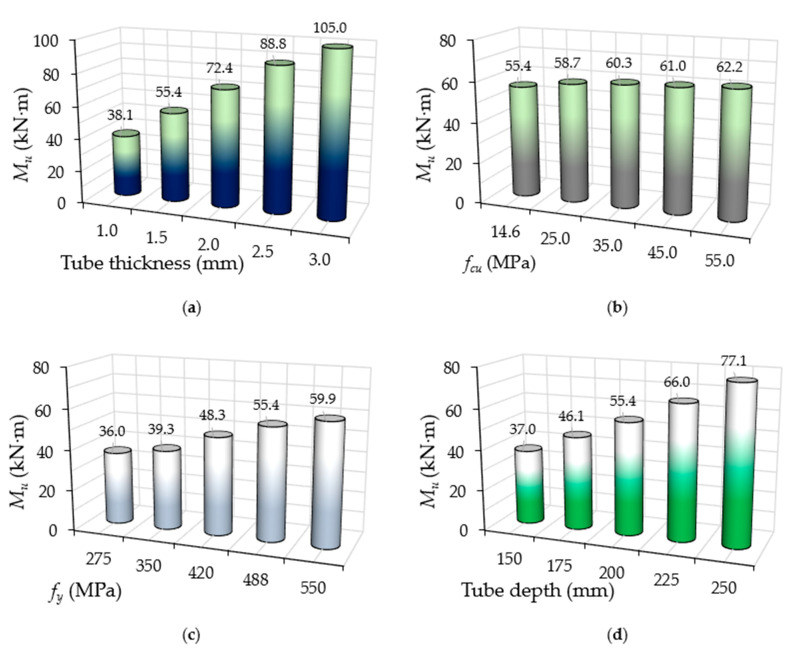
Effects of the *M_u_* values of CFST models with varied parameters: (**a**) group A; (**b**) group B; (**c**) group C; (**d**) group D.

The ultimate bending strength capacities (*M_u_*) of the analysed FE models are given in Table 3. In addition, they are independently compared for each group in Figure 11. Generally, compared to all of the studied parameters, increasing the tube’s thickness (group A) led to major improvements in the suggested CFST models’ *M_u_* values; these improvements were even more significant than the effects of tube’s depth (Group D). Meanwhile, increasing the strength of the concrete infill led to limited improvements in their *M_u_* values. This can be considered a reasonable outcome since the tube’s thickness directly increased the overall area of the steel cross-section, including the internal stiffeners (the lips of the C-sections) at the top and bottom beam’s flanges. Similar outcomes have been recorded in other studies for the conventional CFST beams that were investigated here [10,14,61]. For example, the FB2-A control model with 1.5 mm thickness achieved an *M_u_* value of 55.4 kN·m; this value was increased to about 30.6% (72.4 kN·m) and 60.3% (88.8 kN·m) when only the tube’s thickness was increased to 2.0 mm and 2.5 mm, respectively. Meanwhile, the same *M_u_* value (55.4 kN·m) for the FB1-B model, in which concrete infill of 14.6 MPa strength was used, was increased by about 12.2% (62.2 kN·m) when a three-times-higher compressive strength was used (55 MPa; model FB5-B).

## 5. Design Guidelines

### 5.1. Evaluation of the Obtained Flexural Stiffness

This section evaluates the currently known flexural stiffness values (*K_i_* and *K_s_*) obtained from existing experimental and numerical approaches. The theoretical expressions that are presented in the AIJ-1997 [62], EC4-2004 [63], and ANSI/AISC 360-10 [64] standards are used:

(1) $$K=E_{S}I_{S}+C1E_{C}I_{C}$$

where *E_s_* and *I_s_* are the modulus of elasticity and moment of inertia, respectively, for the steel part. *E_c_* and *I_c_* are the modulus of elasticity and moment of inertia, respectively, for the concrete part. The *C1* is a reduction factor for the concrete stiffness part, which is taken to be 0.6 in EC4-2004, and 0.2 in AIJ-1997. However, in the ANSI/AISC 360-10 standard, the *C1* value is estimated to be 0.6 *+* 2*A_s_/(A_s_ + A_c_)*, but should not exceed 0.9. The *E_c_* value in Equation (1) is 9500 *(f_ck_ +* 8)^1/3^, 4700 *(f_c_)*^0.5^ and 21,000 (*f_c_*/19.6)^0.5^ for the EC4-2004, AISC-2010 and AIJ-1997 standards, respectively. Furthermore, the theoretical methods developed by Han et al. [51] and Al Zand et al. [56] for the independent prediction of the values of flexural stiffness at the initial and serviceability levels (*K_i_* and *K_s_*) were adopted.

**Figure 12:**
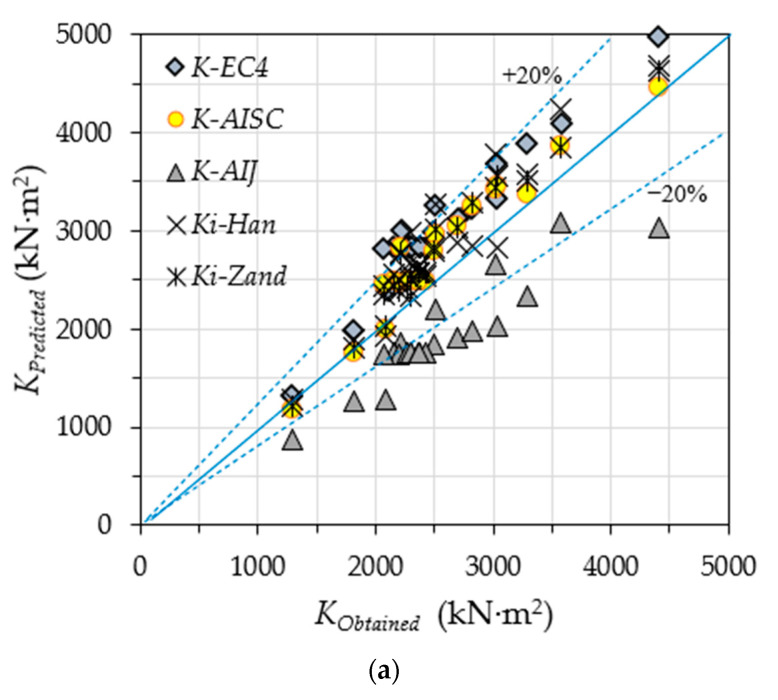

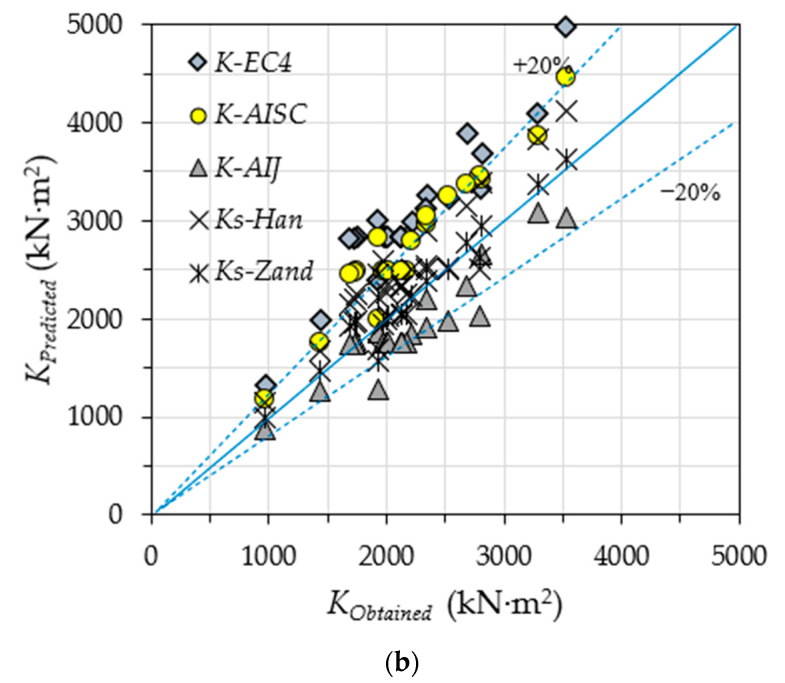
Verification of the obtained flexural stiffness: (**a**) *Ki*; (**b**) *K_s_.*

The predicted values of flexural stiffness (*K_predicted_*), and the experimentally and numerically obtained values (*K_obtained_*), are compared in Figure 12. Generally, the obtained flexural stiffness values at the initial loading stage (*K_i_*) are slightly overestimated by the theoretical prediction, which is acceptable since it is within ±20% [50,52]. However, the AIJ-1997 standard achieves the lowest predicted stiffness values (*K-AIJ*) as compared to the other standards and methods, since this standard uses the lowest concrete stiffness redaction factor (*C1* = 0.2). Additionally, the EC4-2004 and ANSI/AISC 360-10 standards showed a more conservative prediction for the stiffness values (*K-EC4* and *K-AISC*) at the serviceability level (see Figure 12b), given that they use a single-expression formula (Equation (1)) to estimate the flexural stiffness value of the CFST members, unlike the expression methods of Han-2006 and Al Zand-2020, in which the flexural stiffness values of CFST beams are estimated at two different loading stages (two independent levels: *K_i_* and *K_s_*).

### 5.2. Evaluation of the Obtained Flexural Strength

The ultimate flexural strength (*M_u_*) values obtained for the tested and analysed CFST models were evaluated, using the existing theoretical methods given by EC4-2004 [63], Han-2004 [61], and Al Zand-2020 [56], to verify the findings of the current study. In Table 4, the predicted *M_u_* values of the currently investigated beams and models are compared, using the above theoretical methods (*M_u-EC4_*, *M_u-Han_* and *M_u-Zand_*), with those obtained from the current experimental and numerical investigations, including six models analysed by others [19]. Generally, the existing methods showed a conservative prediction of the *M_u_* values of the investigated CFST beams and models, with reasonable coefficient of variation (COV) values, since these methods were mainly developed for conventional CFST beams (section of beams without internal steel stiffeners). In Table 4, it can be seen that *M_u-EC4_* achieved a mean value (MV) of 0.643. However, *M_u-Han_* and *M_u-Zand_* achieved higher MVs, which were 0.740 and 0.734, respectively, since these two methods took into account the effects of the total area of the steel (*A_s_)* of the CFST beam’s cross-section. The above comparison confirmed that the lips of the C-sections used in the Slender CFST beams investigated in this study behaved as internal steel stiffeners, which led to sufficient improvements in their flexural strength capacities [19].

### 5.3. Development of the New Analytical Method

**Figure 13:**
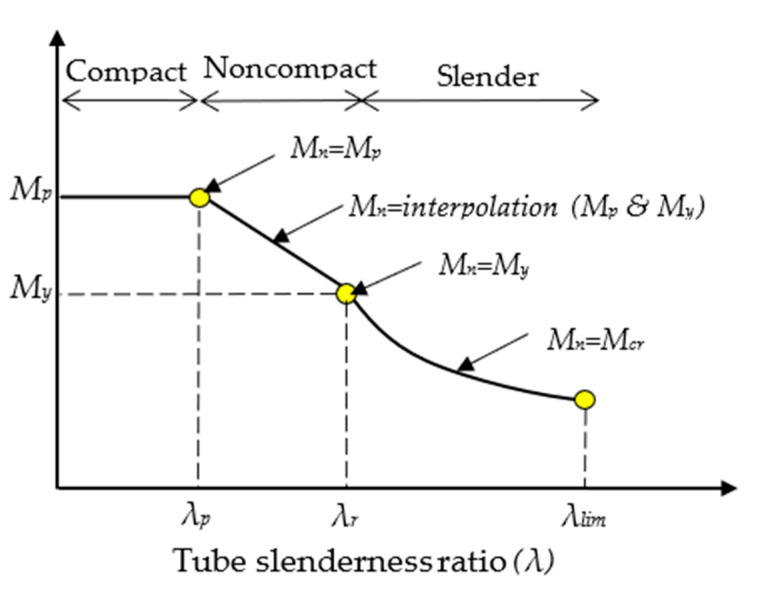
Moment vs. slenderness ratio relationship of the tube’s cross-section.

Generally, the cross-sections of steel tubes are classified into Compact, Noncompact and Slender sections based on their ability to buckle under compression stress. The tube’s effective-width (*W_eff_*)-to-thickness (*t*) ratio, usually known as the slenderness ratio (*λ*), is used as a limit for this classification in the majority of related standardised codes. In the current study, the slenderness limits that are specified in ANSI/AISC 360-10 [64] (Chapter I) were adopted for the classification of the rectangular steel tube beams that were filled with concrete (cross-sections of CFST members). Figure 13 presents the relationship between the nominal flexural strength (Nominal moment; *M_n_*) and the slenderness ratio (*λ*) of the cross-section of CFST beam’s tube. First, the tube’s cross-section was classified as a “Compact section” if the *λ* value was within the limits of the compactness ratio (*λ_p_*), which was equal to 2.26 (*E_s_*/*F_y_*)^0.5^. Second, if the value of *λ* was larger than that of *λ_p_*, but within the limits of the noncompactness ratio (*λ_r_*), which was equal to 3.0 (*E_s_*/*F_y_*)^0.5^, then the tube’s cross-section was classified as a “Noncompact section”. Third, when *λ* exceeded the limit of *λ_r_* but was within the limits of the maximum ratio (*λ_limit_*), which was equal to 5.0 (*E_s_*/*F_y_*)^0.5^, then the cross-section of the CFST beam’s tube was classified as a “Slender section”. This was the case because the ANSI/AISC 360-10 code does not permit the use of Slender CFST beams if their *λ* values exceed the maximum limit (*λ_limit_*).

**Figure 14:**
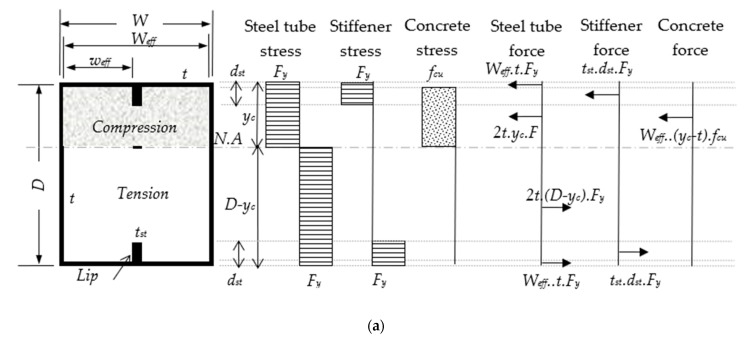

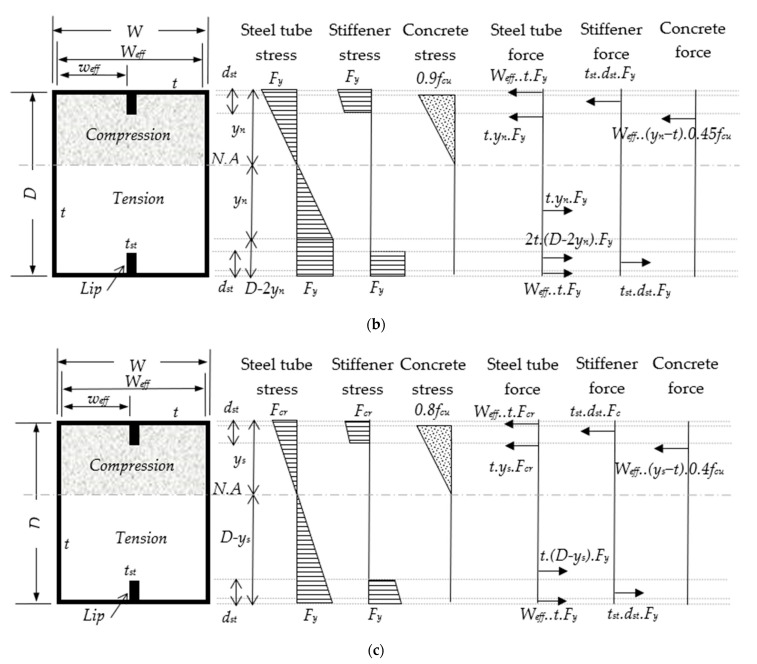
Stress block diagrams of stiffened CFST beam with internal stiffeners: (**a**) compact section; (**b**) non-compact section; (**c**) slender section.

In this study, a novel analytical method for the prediction of the nominal moment (*M_n_*) was developed based on the fundamental theory of stress block diagrams of CFST beams that are internally stiffened with steel stiffeners, as shown in Figure 14. For this purpose, several assumptions were adopted, which are listed as follows:

i. This method was limited to rectangular CFST beams with internal stiffeners under pure static bending.
ii. The shear span-to-depth ratio was assumed to have no significant effects on the CFST beam’s deflection behaviour [51,61].
iii. Full interaction between the concrete infill and the steel tubes/stiffeners was assumed [19,51,61].
iv. In the classification of the stiffened steel tube of the CFST beam, the effective flat width between the web and stiffener (*w_eff_*) was used instead of the overall effective tube width (*W_eff_*) for estimation of the stiffened slenderness ratio (*λ_st_ = w_eff_/t*). In another words, (*λ_st_ = w_eff_/t*) was used instead of (*λ = W_eff_/t*) for the classification.
v. A nominal concrete confinement, which varied considerably based on the slenderness limit, was assumed to have been generated by the steel tube.
vi. The tension-related stress of the concrete, which occurred due to cracking failure, was ignored.
vii. The variations in stress due to the stiffener’s depth and the flange’s thickness were ignored.
viii. Compact section (see Figure 14a): this section was assumed to have rigid-plastic behaviour and the steel stress was assumed to remain within the yielding limit (*F_y_*) at both the tension and compression zones. The concrete compression stress was assumed to be within the limits of the ultimate strength (*f_cu_*) and distributed as a rectangular stress block to the N.A. position.
ix. Noncompact section (see Figure 14b): this section was assumed to have elastic-plastic behaviour at the tension zone and elastic behaviour at the compression zone, and the steel stress was assumed to be within the limits of *F_y_* [12,64]. The concrete compression stress was assumed to be within the limits of 0.9*f_cu_* and distributed as a triangular stress block to the N.A. position.
x. Slender section (see Figure 14c): this section was assumed to have pure elastic behaviour, and the steel stress was assumed to be within the limits of *F_y_* at the maximum tension face and within the limits of the buckling stress (*F_cr_*) at the maximum compression face [12,64]. For this section, a lower concrete compression stress was assumed, which was taken to be within the limits of 0.8*f_cu_*.
xi. Finally, when the forces over the stiffened CFST beam’s cross-section attained equilibrium (see Figure 14), the summarized forms of the new analytical formula for predicting the *M_n_* for each section classification could be expressed as follows:

(2) $$\mathrm{For}\;\mathrm{the}\;\mathrm{Compact}\;\mathrm{section}\;(\lambda_{st}\;\leq \;\lambda_{p})\;y_{c}=({2tDF}_{y}+f_{cu}W_{eff}t)/({4tF}_{y}+f_{cu}W_{eff})\;$$

(3) $$M_{n\;}{=M}_{p}{=W}_{eff}{tF}_{y}\left(D\;-\;t\right){+t}_{\mathrm{st}}d_{\mathrm{st}}F_{y}\left({D\;-\;D}_{\mathrm{st}}\right){+tF}_{y}[y_{c}{}^{2}+\left(D\;-\;y_{c}{)}^{2}\right]+0{.5W}_{eff}f_{cu}{\left(y_{c}\;-\;t\right)}^{2}\;\mathrm{For}\;\mathrm{the}\;\mathrm{Noncompact}\;\sec\mathrm{tion}\;(\lambda_{p}<\lambda_{st}\leq \lambda_{r})$$

(4) $$y_{n}=\left({2tDF}_{y}+0{.45f}_{cu}W_{eff}t\right)/\left({4tF}_{y}+0{.45f}_{cu}W_{eff}\right)\;$$

(5) $$M_{y}{=W}_{eff}{tF}_{y}\left(D\;-\;t\right){+t}_{\mathrm{st}}D_{\mathrm{st}}F_{y}\;\left({D\;-\;D}_{\mathrm{st}}\right){+tF}_{y}D\left(D\;-\;2y_{n}\right){+4/3tF}_{y}y_{n}{}^{2}+0{.3W}_{eff}f_{cu}{\left(y_{n}\;-\;t\right)}^{2}$$

(6) $$M_{n}{=M}_{p}\;-\;\left[\left(M_{p}{-\;M}_{y}\right)\cdot \left(\lambda \;-\;\lambda_{p}\right)/\left(\lambda_{r\;}-\;\lambda_{p}\right)\right]\;\mathrm{For}\;\mathrm{the}\;\mathrm{Slender}\;\sec\mathrm{tion}\;(\lambda_{r}<\lambda_{\mathrm{st}}\leq \lambda_{limit})$$

(7) $$F_{\mathrm{cr}}{=9E}_{s}\;/{\left(W_{eff}/t\right)}^{2}\;$$

(8) $$y_{s}=\left[{tDF}_{y}{+W}_{eff}t\left(0.4f_{cu}{+f}_{y}{\;-\;f}_{\mathrm{cr}}\right){+t}_{\mathrm{st}}d_{\mathrm{st}}\left(F_{y}{-\;f}_{\mathrm{cr}}\right)\right]/\left(t\left(F_{y}{+f}_{\mathrm{cr}}\right)+0.4f_{cu}W_{eff}\right)\;$$

(9) $$M_{n}{=M}_{\mathrm{cr}}{=W}_{eff}{tF}_{\mathrm{cr}}\left(y_{s}\;-t/2\right){+W}_{eff}{tF}_{y}\left(D\;-\;y_{s}\;-\;t/2\right){+t}_{\mathrm{st}}d_{\mathrm{st}}F_{\mathrm{cr}}\left(y_{s}\;{-\;d}_{\mathrm{st}}/2\right)+{\;t}_{\mathrm{st}}d_{\mathrm{st}}F_{y}\left(D\;-\;y_{s}{\;-d}_{\mathrm{st}}/2\right){+2/3tF}_{\mathrm{cr}}y_{s}{}^{2}{+2/3tF}_{y}{\left(D\;-\;y_{s}\right)}^{2}+0{.267W}_{eff}f_{cu}{\left(y_{s}-\;t\right)}^{2}\;$$

Accordingly, the embedded steel stiffeners that were provided to stiffen the sections of the CFST beams/columns significantly reduced the flat distance of their tube’s flanges/walls, which led to a delay in the tube’s buckling failure, as discussed earlier in this paper and previously confirmed in the literature [19,22,47]. On that basis, the classification of the tube sections of the CFST beams could be changed from Slender to Noncompact and/or to Compact due to the influence of these stiffeners, as evidenced in the comparisons between the stiffened slenderness ratios (*λ_st_*) and the *λ* values in Table 4. Furthermore, when compared to the *M_u_* values obtained from the current study and the additional models analysed in [19], the new analytical method achieved the best prediction values (*M_n_*), with MV and COV values of 0.831 and 0.049, respectively, as compared to the existing theoretical methods shown in Table 4.

## 6. Conclusions

The conclusions of the investigated CFST beams are summarized as follows:

➢ The experimental investigation confirmed that the bending capacity of the suggested prefabricated Slender CFST beams made from two pieces of C-sections was enhanced by about 3.7 times even when filled with 70% replacement recycled concrete material.
➢ Under the static bending load, the prefabricated tubular steel beams (double C-sections) filled with recycled concrete (0, 30%, 50%, and 70%) behaved very similarly to the conventional CFST beams. Additionally, the lips of these C-sections were adequately bonded to the concrete and acted as internal stiffeners for the Slender CFST beam’s cross-section, which delayed the outward buckling failures at their top flanges. For example, the use of recycled aggregate to replace raw aggregate at a proportion of up to 70% resulted in slightly lower flexural stiffness and strength capacity values (−7.2% to −10.7%) compared to those obtained using normal concrete.
➢ Generally, it is worth highlighting that the suggested fabricated steel tube beams’ self-weight was increased substantially due to the effect of concrete infill materials. In turn, the flexural strength capacities of these beams were remarkably enhanced, by approximately 409% and 363%, when using normal concrete and 70% recycled concrete mixtures, respectively. These findings are very important from a structural engineering point of view as, regardless the increment of the beams’ self-weight, they can be used to prospectively determine the scenarios in which this composite system would be reliable for the targeted purpose of construction projects. Cost also played a vital role in the construction design process, which was demonstrated when we compared the cost of the concrete and steel sections in the local market.
➢ The flexural behaviour of tested CFST beam was accurately simulated using the ABAQUS software. The results obtained from the non-linear analyses of FE CFST models that were prepared for investigation of various parameters confirmed that slightly increasing the thickness of the tubes in these models had a major influence on their flexural strengths and stiffnesses as compared to the effects of other parameters. In contrast, a limited degree of influence was achieved when the compressive strengths of the concrete infill material and/or the yielding strengths of the steel tubes were increased.
➢ Lastly, the newly developed analytical method achieved the best prediction of the flexural strength capacities of the internally stiffened CFST beams that were tested and analysed in this study, since it was able to independently consider the influence of internal steel stiffeners along with the effects of the properties of steel tubes and concrete. However, this method was found to only be reliable for the internally stiffened rectangular CFST beams.

It is worth highlighting the main research limitation of the current investigation, namely that the uncertainties related to this model could be further examined as they are very significant in terms of the parameters of structural behavior. In addition, further experimental/numerical investigations, other than the rectangular cross-sections under static/dynamic loading scenarios, could be conducted on the stiffened CFST beams.

## Figures and Tables

**Table 1:** Designations and results of the tested specimens.

Specimen Designations	*D* × *B* × *t* (mm)	*L_e_* (m)	*f_y_* (MPa)	*f_cu_* (MPa)	*E_c_* (GPa)	*M_u_* (kN·m)	*K_i_* (kN·m^2^)	*K_s_* (kN·m^2^)	EAI (kN·mm)
HB	200 × 150 × 1.5	2.8	489	-	-	14.1	1207	1121	201.8
FB-RC0	200 × 150 × 1.5	2.8	489	26.2	21.1	57.7	2216	1924	5996
FB-RC30	200 × 150 × 1.5	2.8	489	14.6	14.5	53.7	2157	1751	5539
FB-RC50	200 × 150 × 1.5	2.8	489	14.1	13.8	52.6	2194	1726	5493
FB-RC70	200 × 150 × 1.5	2.8	489	13.7	13.2	51.5	2066	1690	5439

**Table 2:** Proportions of the concrete mixtures (kg/m^3^).

Mixture Designations	Cement	Fine Agg.	Coarse Agg.	Silica Fume	EPS (%)	EPS	RAC (%)	RAC	Water	Slump (mm)	Density
RC0	390	700	1115	-	-	-	-	-	180	128	2295
RC30	350	700	781	40	30	2.1	-	-	180	147	1881
RC50	350	700	558	40	30	2.1	20	190	180	151	1813
RC70	350	700	335	40	30	2.1	40	380	180	154	1772

**Table 3:** FE model designations and analysis results.

Models Designation	*D × B × t* (mm)	*f_y_* (MPa)	*f_cu_* (MPa)	*E_c_* (GPa)	*As* (mm^2^) *×*10^2^	*Is* (mm^4^) *×*10^6^	*Ac* (mm^2^) *×*10^4^	*Ic* (mm^4^) *×*10^7^	*M_u_* (kN·m)	*K_i_* (kN·m^2^)	*K_s_* (kN·m^2^)
FB1-A	200 × 150 × 1.0	489.0	14.6	16.2	7.72	4.87	2.92	9.52	38.1	2093	1929
^#^ FB2-A	200 × 150 × 1.5	489.0	14.6	16.2	11.5	7.23	2.88	9.28	55.4	2375	2125
FB3-A	200 × 150 × 2.0	489.0	14.6	16.2	15.3	9.54	2.85	9.05	72.4	2511	2345
FB4-A	200 × 150 × 2.5	489.0	14.6	16.2	19.0	11.8	2.81	8.82	88.8	3024	2815
FB5-A	200 × 150 × 3.0	489.0	14.6	16.2	22.7	14.0	2.77	8.60	105.0	3575	3285
^#^ FB1-B	200 × 150 × 1.5	489.0	14.6	16.2	11.5	7.23	2.88	9.28	55.4	2375	2125
FB2-B	200 × 150 × 1.5	489.0	25.0	21.2	11.5	7.23	2.88	9.28	58.7	2491	2219
FB3-B	200 × 150 × 1.5	489.0	35.0	25.0	11.5	7.23	2.88	9.28	60.3	2699	2339
FB4-B	200 × 150 × 1.5	489.0	45.0	28.4	11.5	7.23	2.88	9.28	61.0	2819	2529
FB5-B	200 × 150 × 1.5	489.0	55.0	31.4	11.5	7.23	2.88	9.28	62.2	3033	2798
FB1-C	200 × 150 × 1.5	275.0	14.6	16.2	11.5	7.23	2.88	9.28	36.0	2293	1978
FB2-C	200 × 150 × 1.5	350.0	14.6	16.2	11.5	7.23	2.88	9.28	39.3	2274	1998
FB3-C	200 × 150 × 1.5	420.0	14.6	16.2	11.5	7.23	2.88	9.28	48.3	2302	2011
^#^ FB4-C	200 × 150 × 1.5	489.0	14.6	16.2	11.5	7.23	2.88	9.28	55.4	2375	2125
FB5-C	200 × 150 × 1.5	550.0	14.6	16.2	11.5	7.23	2.88	9.28	59.9	2432	2173
FB1-D	150 × 150 × 1.5	489.0	14.6	16.2	10.0	3.74	2.15	3.85	37.0	1287	978
FB2-D	175 × 150 × 1.5	489.0	14.6	16.2	10.8	5.32	2.52	6.17	46.1	1814	1436
^#^ FB3-D	200 × 150 × 1.5	489.0	14.6	16.2	11.5	7.23	2.88	9.28	55.4	2375	2125
FB4-D	225 × 150 × 1.5	489.0	14.6	16.2	12.3	9.51	3.25	13.3	66.0	3286	2686
FB5-D	250 × 150 × 1.5	489.0	14.6	16.2	13.0	12.2	3.62	18.3	77.1	4407	3530

^#^ Verified FE model with the corresponding tested FB-RC30 specimen.

**Table 4:** Verification of the obtained *M_u_* values of the tested specimens and analysed FE models.

Model Designations	*λ* *(W/t)*	*λ_st_* *(W_eff_/t)*	*M_u_* (kN.m)	*M_u-EC4_* (kN.m)	*M_u-EC4_* /*M_u_*	*M_u-Han_* (kN·m)	*M_u-Han_* */M_u_*	*M_u-P1_* (kN·m)	*M_u-P1_* */M_u_*	*M_n_* (kN·m)	*M_n_* */M_u_*
FB-RC0	98.0	48.0	57.7	39.1	0.678	42.3	0.732	42.6	0.738	48.9	0.848
FB-RC30	98.0	48.0	53.7	37.3	0.694	39.3	0.731	37.2	0.692	46.7	0.870
FB-RC50	98.0	48.0	52.6	37.2	0.706	39.2	0.745	36.8	0.700	46.6	0.886
FB-RC70	98.0	48.0	51.5	37.1	0.720	39.1	0.759	36.5	0.709	46.5	0.903
FB1-A	148.0	73.0	38.1	25.8	0.678	27.2	0.713	27.1	0.711	30.1	0.789
FB2-A	98.0	48.0	55.4	37.2	0.671	39.2	0.707	37.1	0.670	46.7	0.842
FB3-A	73.0	35.5	72.4	48.2	0.665	51.7	0.714	48.6	0.671	61.4	0.848
FB4-A	58.0	28.0	88.8	58.8	0.663	64.8	0.730	61.7	0.695	75.1	0.846
FB5-A	48.0	23.0	105.0	69.3	0.660	78.5	0.747	68.0	0.647	88.6	0.844
FB1-B	98.0	48.0	55.4	37.2	0.671	39.2	0.707	37.1	0.670	46.7	0.842
FB2-B	98.0	48.0	58.7	38.9	0.663	41.9	0.713	42.1	0.717	48.6	0.829
FB3-B	98.0	48.0	60.3	40.0	0.663	44.5	0.738	45.5	0.755	49.9	0.827
FB4-B	98.0	48.0	61.0	40.8	0.669	46.9	0.769	49.3	0.809	50.7	0.832
FB5-B	98.0	48.0	62.2	41.4	0.666	49.1	0.789	53.7	0.863	51.4	0.826
FB1-C	98.0	48.0	36.0	22.0	0.611	23.7	0.660	23.9	0.665	27.8	0.772
FB2-C	98.0	48.0	39.3	27.4	0.697	29.1	0.741	28.9	0.735	34.7	0.883
FB3-C	98.0	48.0	48.3	32.4	0.671	34.2	0.708	33.2	0.688	41.1	0.851
FB4-C	98.0	48.0	55.4	37.2	0.671	39.2	0.707	37.1	0.670	46.7	0.842
FB5-C	98.0	48.0	59.9	41.5	0.693	43.8	0.731	40.4	0.674	51.1	0.853
FB1-D	98.0	48.0	37.0	24.5	0.662	25.4	0.688	23.4	0.632	31.0	0.839
FB2-D	98.0	48.0	46.1	30.6	0.663	32.0	0.694	29.9	0.649	38.5	0.836
FB3-D	98.0	48.0	55.4	37.2	0.671	39.2	0.707	37.1	0.670	46.7	0.842
FB4-D	98.0	48.0	66.0	44.4	0.672	47.1	0.713	45.0	0.682	55.4	0.839
FB5-D	98.0	48.0	77.1	52.1	0.676	55.7	0.722	53.6	0.695	64.8	0.840
^#^ SB2-SI (St1.5)	131.3	65.2	60.1	35.0	0.584	45.4	0.755	53.1	0.885	43.2	0.719
^#^ SB2-SI (St3.0)	131.3	64.7	64.2	35.3	0.550	51.3	0.799	56.8	0.884	48.1	0.749
^#^ SB2-SI (St4.5)	131.3	64.2	69.7	35.6	0.510	57.2	0.820	60.9	0.873	53.6	0.768
^#^ SB3-DI (St1.5)	131.3	43.1	65.5	35.3	0.539	50.9	0.777	56.5	0.863	50.3	0.769
^#^ SB3-DI (St3.0)	131.3	42.4	76.5	35.8	0.468	63.0	0.823	65.4	0.854	62.4	0.816
^#^ SB3-DI (St4.5)	131.3	41.8	88.0	36.4	0.413	74.6	0.848	75.3	0.856	76.6	0.870
MV	-	-	-	-	0.643	-	0.740	-	0.734	-	0.831
COV	-	-	-	-	0.112	-	0.057	-	0.110	-	0.049

^#^ FE CFST models stiffened with internal I-steel stiffeners analysed by Al Zand et al. [19].

## Data Availability

Data are presented in the article.

## Abbreviations

Area of concrete core cross-section (*A_c_*), area of steel tube cross-section (*As)*, coefficient of variation (COV), depth of rectangular steel tube (*D*), depth of steel stiffener (*d_st_*), modulus of elasticity for concrete (*E_c_*), modulus of elasticity for steel (*E_s_*), compressive strength of concrete cube at 28 days (*f_cu_*), characteristic concrete strength of 0.67*f_cu_* (*f_ck_*), ultimate strength of steel (*f_u_*), yield strength of steel (*f_y_*), steel tube buckling stress (*F_cr_*), moment of inertia for concrete tube cross-section (*I_c_*), moment of inertia for steel tube cross-section (*I_s_*), initial flexural stiffness of composite section (*K_i_*), serviceability level of the flexural stiffness of the composite section (*K_s_*), effective length of the specimen (*M*), ultimate bending moment capacity (flexural strength capacity; *M_u_*), nominal bending moment (*M_n_*), plastic limit bending moment (*M_p_ = M_u_*), yield limit bending moment (*M_y_*), mean value (*MV*), steel tube/C-section thickness (*t*), steel stiffener thickness (*t_st_*), width of rectangular steel tube (*W*), effective width of rectangular steel tube (*W_eff_*), effective width/distance between two internal stiffeners and/or between the edge of the tube and the first internal stiffener (*w_eff_*), distance of N.A. from the top flange of the tube for the Compact section (*y_c_*), distance of N.A. from the top flange of the tube for the Noncompact section (*y_n_*), distance of N.A. from the top flange of the tube for the Slender section (*y_s_*), slenderness ratio of steel tube cross-section (*λ*), slenderness ratio at the compactness limit (*λ_p_*), slenderness ratio at the noncompactness limit (*λ_r_*), maximum limit of the slenderness ratio (*λ_limit_*), stiffened slenderness ratio (*λ_st_ = w_eff_/t*).
